# 920. Automated Hepatitis C Screening and Linkage to Care among Hospitalized Patients Born Between 1945-1965

**DOI:** 10.1093/ofid/ofab466.1115

**Published:** 2021-12-04

**Authors:** Julia A Gasior, Rebecca Russell, Vincent Lo Re, Anne Norris, Schenevelyn Bennett, Nancy Aitcheson, Nicole Ferrante, Chalanda Evans, Mitesh Patel, Shivan Mehta, jessie Torgersen

**Affiliations:** 1 Perelman School of Medicine, Philadelphia, Pennsylvania; 2 University of Pennsylvania Health System, Philadelphia, Pennsylvania; 3 University of Pennsylvania, Philadelphia, PA; 4 Penn Presbyterian Medical Center, Philadelphia, Pennsylvania; 5 Perelman School of Medicine, University of Pennsylvania Health System, Philadelphia, Pennsylvania

## Abstract

**Background:**

Hepatitis C virus (HCV) infects 4.1 million people in the United States, of whom 50% are unaware of their status. In 2016, Pennsylvania introduced a law mandating HCV screening for patients born between 1945-1965 in inpatient settings. However, HCV screening during hospital admissions has remained low in part due to limited knowledge on HCV testing requirements, interpretation of results, and treatment approaches. To overcome these barriers, we implemented a quality improvement initiative to automate HCV screening as part of hospital admission order sets, facilitate linkage to HCV treatment, and sought to evaluate its effectiveness.

**Methods:**

Between September 2020 and May 2021, the automated inpatient HCV screening strategy was implemented at a single 328-bed academic hospital in Philadelphia, PA. Patients born between 1945-1965 without documentation of HCV screening or diagnosis in the electronic medical record had a HCV antibody with reflexive confirmatory RNA assay automatically populated in the admission order set. Admitting providers could opt out of the screening as appropriate. All patients with reactive HCV antibody were approached by the Hepatitis Linkage Team for result disclosure, counseling, and linkage to treatment for those with HCV viremia. Cascade of care was detailed for those linked to providers within the health system.

**Results:**

During the initial 8 months of the program, 2,203 patients were screened for HCV, identifying 156 with reactive HCV antibody (7.1% seroprevalence). Among 147 with completed HCV RNA assay, 51 were viremic (34.7%). Fourteen viremic patients were not linked to care, including six with a terminal illness, two who declined linkage, and six who did not respond to linkage attempts. Nine were linked to care at other health systems. Among the 28 patients linked to providers in the health system, 50% completed initial visits, 42.8% were prescribed direct acting antivirals (DAA), and 21.4% completed therapy by May 2021. One person achieved sustained virologic response 12 weeks after treatment as of May 2021 (**Figure 1**).

Figure 1. Cascade of HCV Care Among Patients Screened During Hospital Admission from September 2020 to May 2021

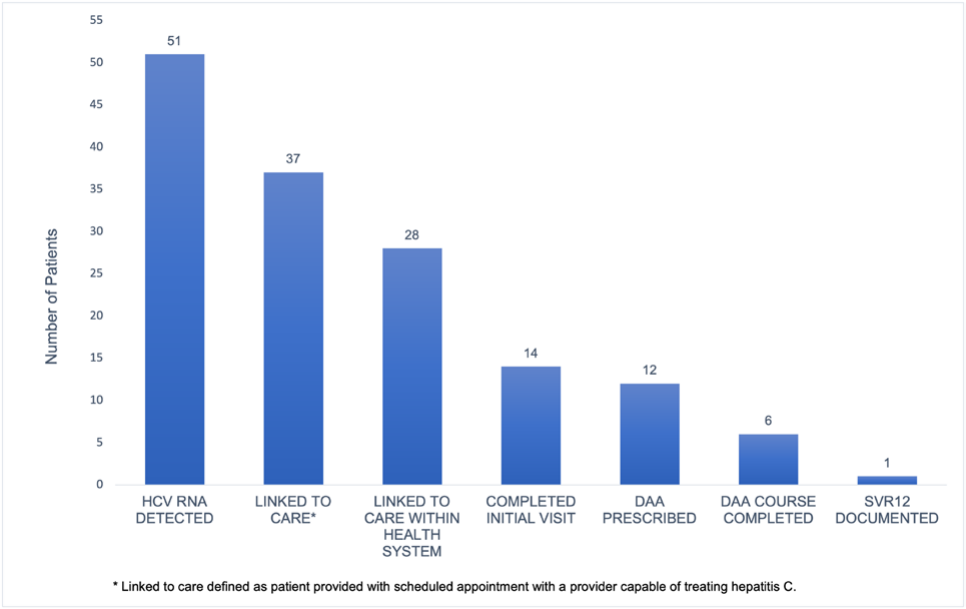

**Conclusion:**

Automated inpatient HCV screening is a viable strategy to identify people with HCV and facilitate linkage to care. Optimal strategies to ensure patients access and maintain care require further study.

**Disclosures:**

**All Authors**: No reported disclosures

